# Label-free proteomic analysis reveals the hepatoprotective mechanism of gypenosides in liver injury rats

**DOI:** 10.3389/fphar.2024.1417575

**Published:** 2024-06-27

**Authors:** Yu Chen, Lizhou Ma, Yibo Wang, Jiarui Zhang, Tianhe Pei, Miao Wang

**Affiliations:** ^1^ School of Life Science and Biopharmaceutics, Shenyang Pharmaceutical University, Shenyang, China; ^2^ School of Pharmacy, Shenyang Pharmaceutical University, Shenyang, China; ^3^ Faculty of Functional Food and Wine, Shenyang Pharmaceutical University, Shenyang, China; ^4^ School of Traditional Chinese Materia Medica, Shenyang Pharmaceutical University, Shenyang, China

**Keywords:** proteomics, liver injury, gypenosides, label-free, parallel reaction monitoring

## Abstract

Chronic liver disease, a long-term condition resulting from various causes such as alcohol abuse, metabolic disorders, and viral hepatitis, is becoming a significant global health challenge. Gypenosides (GPs), derived from the traditional Chinese medicine *Gynostemma pentaphyllum* (Thunb.) Makino, exhibited hepatoprotective properties in recent years, yet the precise therapeutic mechanism remains unclear. In this study, label-free and parallel reaction monitoring (PRM) proteomics were used to elucidate the hepatoprotective mechanism of GPs in liver injury rats. Through label-free proteomics, we identified 2104 differentially expressed proteins (DEPs) associated with liver injury, along with 1974 DEPs related to the effects of GPs. Bioinformatics analysis revealed that GPs primarily restored metabolic processes involving valine, leucine, and isoleucine degradation, as well as propanoate and butanoate metabolism, and steroid hormone biosynthesis during liver injury. Subsequently, overlapping the two groups of DEPs identified 1508 proteins reversed following GPs treatment, with key targets further validated by PRM. Eight target proteins were identified for GPs treatment of liver injury, including Lgals3, Psat1, Phgdh, Cyp3a9, Cyp2c11, Cyp4a2, Glul, and Ces1d. These findings not only elucidated the hepatoprotective mechanism of GPs, but may also serve as potential therapeutic targets of chronic liver disease.

## 1 Introduction

Chronic liver disease stands as a significant global health concern, primarily attributed to chronic hepatitis B virus (HBV), hepatitis C virus (HCV), alcohol-related liver disease (ALD), and non-alcoholic fatty liver disease (NAFLD). These conditions often lead to cirrhosis and liver cancer, resulting in approximately two million fatalities annually worldwide. (Moon et al., 2020; Devarbhavi et al., 2023). To investigate the mechanism of liver injury and unearth effective therapeutic interventions, researchers often turn to the carbon tetrachloride (CCl_4_)-induced chronic liver disease model in rats due to its reliablility and effectiveness (Qin et al., 2012; Fortea et al., 2018; Zahira et al., 2023). However, despite advancements in liver disease research, there is still a lack of effective treatments.

In recent years, research on the application of traditional Chinese medicine in the treatment of liver diseases has gained considerable attention owing to its multi-component and multi-target characteristics. *Gynostemma pentaphyllum* (Thunb.) Makino is a traditional Chinese medicine commonly used in the treatment of hyperlipidemia and fatty liver disease. Gypenosides (GPs), a saponin extract from *Gynostemma pentaphyllum* (Thunb.) Makino has demonstrated hepatoprotective effects through diverse mechanisms (Chen et al., 2000). Metabolomic and proteomic investigations have revealed the anti-hepatic fibrosis effects of GPs, highlighting associations with glycolytic pathways and ALDH protein targets (Song et al., 2017). Our previous study illustrated GPs’ ability to modulate metabolites levels and restore gut microbiota, thereby attenuating CCl_4_-induced liver injury (Tian et al., 2022). However, most previous studies predominantly focused on the limited metabolites and protein targets of GPs, and have lacked high-throughput validation of their protein targets.

Label-free quantitative proteomics provides the advantages of not requiring isotopic or chemical labeling, allowing for greater analytical depth, higher dynamic range, and larger proteome coverage (Bantscheff et al., 2007). Accordingly, the technique has been used in several studies to investigate molecular mechanisms and pathways, leading to the identification of biomarkers or therapeutic targets for a variety of diseases (Corrêa et al., 2017; Zhi et al., 2022). Parallel reaction monitoring (PRM) is a proteomics method based on high-resolution hybrid mass spectrometry for high-selectivity quantification of target proteins and target peptides (Rauniyar, 2015). PRM can identify multiple target proteins simultaneously, compared to classic validation methods such as Western blotting and ELISA. These advantages make it a useful method for proteomics validation. In this study, we investigated the differentially expressed proteins (DEPs) in liver injury rats after GPs treatment by using label-free technology for protein profiling, and determined the functions of these proteins by bioinformatics analysis, and finally found the key protein targets by PRM validation.

## 2 Methods

### 2.1 Chemical reagents

Gypenosides tablet (batch number: B1803274) was purchased from Guangzhou Baiyunshan Hutchison Whampoa Traditional Chinese Medicine Co., Ltd. (Guangzhou, China); Formaldehyde solution (analytical grade) was purchased from Wuxi Yasheng Chemical Co., Ltd. (Jiangsu, China); Sodium Chloride Injection was purchased from Zhejiang Tianli Pharmaceutical Co., Ltd. (Zhejiang, China); Alanine amino Transferase (ALT) and aspartate aminotransferase (AST) kits were purchased from Nanjing Jiancheng Bioengineering Institute (Nanjing, China).

### 2.2 Animal experiments

Fifteen male Sprague-Dawley (SD) rats (4-week-old, 200 ± 20 g) were supplied by the Experimental Animal Center of Shenyang Pharmaceutical University (Shenyang, China). These animals were maintained in an environmentally controlled room with a 12/12 light-dark cycle, a temperature range of 20°C–25°C, a relative humidity of 40%–70%, and unrestricted access to food and water. All animal protocols used in this study were approved by the Medical Ethics Committee of Shenyang Pharmaceutical University and were in accordance with the guidelines of the National Institutes of Health on Animal Care.

All animals were randomly divided into three groups of five each after a week of acclimation: the control group (CON group), the liver injury group (MOD group), and the group receiving GPs therapy (GPs group). To induce liver injury in rats, the MOD and GPs groups were given 40% (v/v) CCl_4_ solution in soybean oil (2 mL/kg) via oral administration twice a week for 6 weeks, while the CON rats received the same amount of soybean oil by the same method (Zhang et al., 2021a). Upon successful establishment of the liver injury model, the rats in the GPs group were administered GPs at a dose of 200 mg/kg once daily for 8 weeks (Song et al., 2017; Tian et al., 2022), while the rats in the CON and MOD groups were administered an equivalent amount of normal saline. To avoid any potential recovery effects of liver, the MOD and GPs groups continued to receive the 40% CCl_4_ soybean oil solution (2 mL/kg) once weekly throughout the six-week treatment.

### 2.3 Sample collections and preparation

Rats underwent fasting overnight prior to blood collection from their orbital venous plexus at the end of the 14th week. The collected blood was transferred to heparinized tubes and centrifuged at 1,100 *g* for 15 min to separate the plasma. Subsequently, the rats were euthanized and their liver and spleen were collected. The organs were weighed, and organ indices were calculated based on their percentage of body weight. Plasma and liver tissues were stored at −80°C for subsequent analysis.

### 2.4 Assessment of liver injury

The serum concentrations of ALT and AST were determined using commercial kits, following the manufacturer’s instructions. Liver tissue samples were fixed in 10% formalin solution for 24–48 h at room temperature embedded in paraffin, and sectioned into 5 µm thick slices. The tissue sections were then stained with hematoxylin and eosin, examined under a light microscope at ×400 magnification, and images were captured for analysis.

### 2.5 Protein extraction and trypsin digestion

Liver tissues were ground into a powder using liquid nitrogen, transferred to a 5 mL centrifuge tube, and sonicated on ice in four volumes of lysis buffer (8 M urea and 1% Protease Inhibitor Cocktail). The remaining debris was removed through centrifugation at 10,000 g for 10 min at 4 °C, and the protein concentration was assessed using a BCA kit (Beyotime, China).

Each sample protein was digested with trypsin, and a precipitate was formed by adding 20% trichloroacetic acid (TCA) and mixing it with vortex. The precipitate was then centrifuged at 4,500 *g* for 5 min at 4 °C, the supernatant was discarded, and washed with pre-cooled acetone three times. Then, 200 mM TEAB was added and sonicated to disperse. The protein was digested overnight with trypsin at a ratio of 1:50 (protease: protein, m/m). The solution was then reduced with dithiothreitol (DTT) to a final concentration of 5 mM for 30 min at 56°C. Finally, iodoacetamide (IAA) was added to a final concentration of 11 mM, and the solution was incubated for 15 min at room temperature in the dark.

### 2.6 LC-MS/MS analysis for label-free quantitative proteomics

Tryptic peptides were dissolved in 0.1% formic acid and 2% acetonitrile (solvent A) and directly placed on an Agilent 300Extend C18 column (250 mm, 4.6 mm, 5 m; Agilent, United States). The gradient consisted of an increase from 6% to 23% solvent B (0.1% formic acid in 98% acetonitrile) over 68 min, 23%–32% over 14 min, 80% over 4 min, and 80% over the last 4 min, all at a constant flow rate of 500 nL/min using an EASY-nLC 1000 UPLC system.

The peptides were analyzed using an NSI source, followed by tandem mass spectrometry (MS/MS) in a Q Exactive™ Plus (Thermo Fisher Scientific) linked online to the UPLC. The electrospray voltage was set to 2.3 kV, and the FAIMS compensation voltages (CV) were −45 V and −65 V. The m/z scan range for the full scan was 400–1,200 and intact peptides were detected at a resolution of 60,000. Peptides were then selected for MS/MS analysis with the NCE set to 27, and fragments were detected at a resolution of 15,000. A data-dependent acquisition approach was used, in which an MS scan was followed by 25 MS/MS scans. The AGC was set to 5E4 ions/s and the maximum IT was set to Auto.

The raw data were retrieved using Proteome Discoverer (v2.4.1.15). The database was Rattus_norvegicus_10116_PR_20210721. fasta (29,934 sequences). Retrieve parameters: enzyme was set as Trypsin (Full), max missed cleavage set as 2. The minimum peptide length was set to 6. Variable modifications were set as oxidation (M), acetyl (N-terminus), met-loss (M), and met-loss + acetyl (M), and maximum variable modifications were set as 3. The precursor mass tolerance was set at 10 ppm, and the product mass tolerance was set at 0.02 Da. The FDR for protein and PSM identification was set at 1%.

### 2.7 Bioinformatics analysis

The relative quantitative values of each protein (Log2 transformed) were subjected to a Student’s t-test. Proteins exhibiting a fold change (FC) of more than 1.5 or less than 0.67 (*p* < 0.05) were identified as DEPs. The DEPs were visualized using volcano plots and heatmaps generated with the R packages “ggplot2” and “pheatmap.“The procedure of protein annotation was as previously described (El-Rami and Sikora, 2019). The Protein annotation was performed as previously described. Gene Ontology (GO) annotation proteome was derived from the UniProt-GOA database (http://www.ebi.ac.uk/GOA/). First, the identified protein ID was converted to a UniProt ID and then mapped to GO IDs by protein ID. Proteins were then classified by GO annotation into three categories: biological processes (BP), molecular functions (MF), and cellular components (CC), using the eggnog-mapper v2.0. Kyoto Encyclopedia of Genes and Genomes (KEGG) database was used to annotate the protein pathways. First, the KEGG online service tool KAAS (http://www.genome.jp/kaas-bin/kaas_main) was used to annotate the protein’s KEGG database description. Then mapping the annotation result on the KEGG pathway database using KEGG online service tools KEGG mapper (http://www.kegg.jp/kegg/mapper.html). Subcellular localization annotation of proteins was performed using the WolF Psort software (https://wolfpsort.hgc.jp/).

For enrichment analysis, KEGG database and Pfam database were used for pathway enrichment analysis and functional structural domain enrichment analysis of differentially expressed proteins, respectively.

### 2.8 Validation of DEPs by PRM

Tryptic peptides were separated using an EASY-nLC 1000 UPLC system. Mobile phases A and B were identical to those used in the label-free experiment. The gradient settings: 0–40 min, 6%–20% B; 40–52 min, 20%–30% B; 52–56 min, 30%–80% B; 56–60 min, 80% B, with a flow rate of 500 nL/min.

The raw data were retrieved using Maxquant (v1.6.15.0). Rattus_norvegicus_10116_PR_20210721. fasta (29,934 sequences). Retrieve parameters: enzyme was set as Trypsin/P, max missed cleavage was set as 2. The minimum peptide length was set as 7. A fixed modification was set as cysteine alkylation carbamidomethyl (C); variable modifications were set as oxidation (M), acetyl (N-terminus), and decarboxamidation (NQ); and maximum variable modifications were set at 5. The precursor mass tolerances of the first and main searches were set at 20 ppm and 4.5 ppm, respectively. The product mass tolerance was set to 20 ppm. The FDR for protein and PSM identification was set at 1%.

Skyline v.3.6 software was used to process the generated MS data. Peptide settings: Trypsin [KR/P] enzyme, max missed cleavage set to 2. The peptide length was set to 7–25 and the variable modification was to Carbamidomethyl on Cys. Transition settings: precursor charges were set to 2 and 3, ion charges were set to 1, and ion types were set to b and y. The product ions were set from ion 3 to the last ion, with an ion-match tolerance of 0.02 Da.

### 2.9 Statistical analysis

The results of ALT, AST, liver index, and spleen index in the liver injury assessment were analyzed using Student’s t-test with SPSS 19.0 (Chicago, IL, United States). For the screening of DEPs and generating their volcano plots and heatmaps, a *t*-test was also employed. To ensure that the data conformed to a normal distribution, the relative protein quantification values were Log2 transformed. Fisher’s exact test was used for GO, KEGG, and protein domain enrichment analyses, along with their bubble plots. In all statistical analysis, *p* < 0.05 was considered statistically significant.

## 3 Manuscript formatting

### 3.1 Assessment of CCl_4_-induced liver injury

**FIGURE 1:**
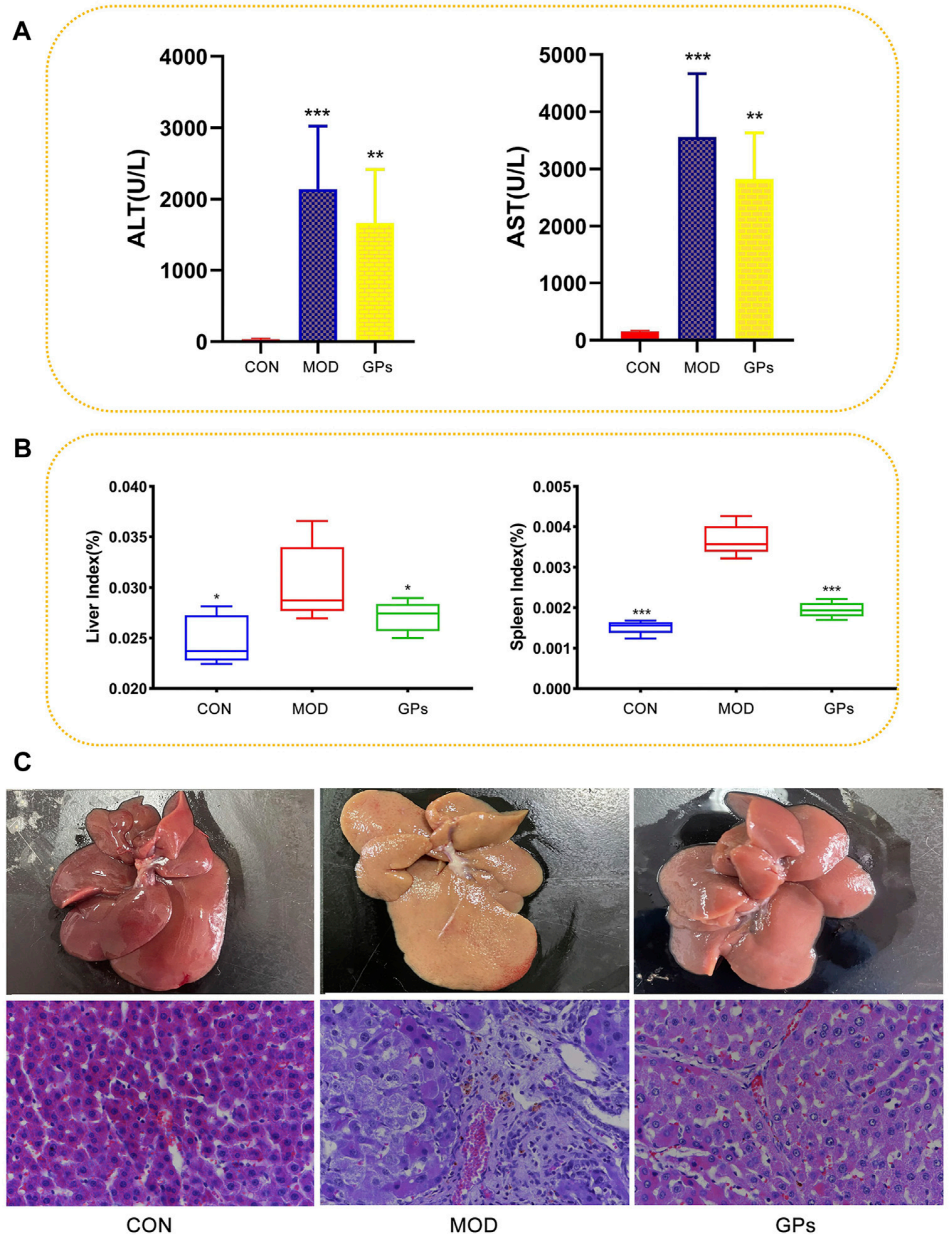
Effect of GPs on the CCl_4_-induced liver injury (n = 5). **(A)** ALT and AST in the plasma samples, ***p < 0.001, **p < 0.01 compared with the MOD group; **(B)** Liver and spleen indices, ***p < 0.001, *p < 0.05 compared with the MOD group; **(C)** HE staining of rat liver tissue. Magnification: ×400.

After six-week CCl_4_ treatment, serum levels of ALT and AST significantly increased, confirming the successful establishment of a liver injury model (Figure 1A). Compared to the CON group, the MOD group showed higher liver and spleen indices, which were reversed after GPs treatment (Figure 1B). H&E staining results revealed that the liver structure in the control group was normal, with clear hepatic sinus morphology. In contrast, the MOD group showed signs of liver damage, with evident inflammatory cell infiltration and vacuole-like lesions in the portal area. Compared to the MOD group, the GPs group showed a decrease in vacuole-like lesions and significant liver injury reduction (Figure 1C). These findings indicate that GPs possess therapeutic properties in CCl_4_-induced liver injury.

### 3.2 Quantification of identified proteins

A total of 746,132 spectra matching the theoretical secondary spectra were identified after scanning the database of 1,658,625 secondary spectra obtained by mass spectrometry. Spectral analysis identified 49,418 peptides, 45,373 of which were unique. A total of 5,847 proteins were identified by unique peptides, of which 4,836 were quantified (Supplementary Figure S1A).

**FIGURE 2:**
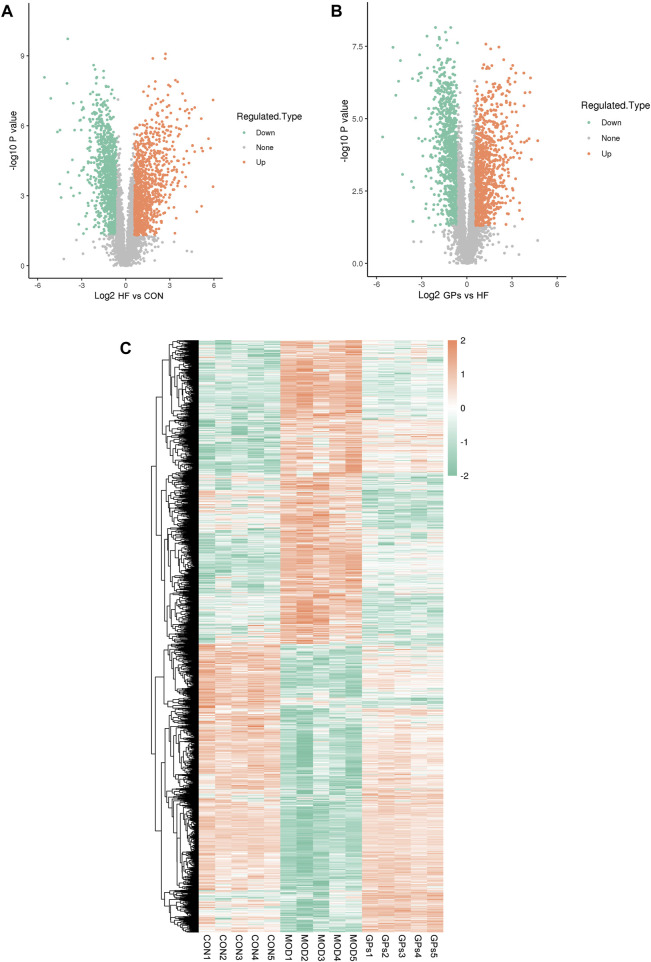
Proteomic features of DEPs in CCl_4_-induced liver injury. **(A)** Volcano plot for DEPs between MOD and CON groups; **(B)** Volcano plot for DEPs between GPs and MOD groups; **(C)** Heatmap of DEPs in CCl_4_-induced liver injury.

Compared with the CON group, 2,109 proteins exhibited significant alterations in the MOD group, with 1,037 upregulated and 1,072 downregulated (Figure 2A). Comparatively, between the MOD and GPs groups, 1,974 proteins were significantly different, with 986 upregulated and 988 downregulated proteins (Figure 2B). The comprehensive lists of DEPs are provided in Supplementary Tables S1, S2. A heatmap illustrated 1,516 co-differential proteins: 687 DEPs were increased in the MOD group and decreased following GPs treatment, while 821 DEPs were decreased in the MOD group and were reversed in the GPs group (Figure 2C).

Statistical analysis methods, including Pearson’s correlation coefficient, principal component analysis (PCA), and relative standard deviation (RSD), were used to assess the quantitative reproducibility of proteins. The results revealed that the quantitative results of the biological replicates or technical replicates were statistically consistent (Supplementary Figures S1B–S1D).

### 3.3 Bioinformatics analysis of DEPs

#### 3.3.1 GO analysis

**FIGURE 3:**
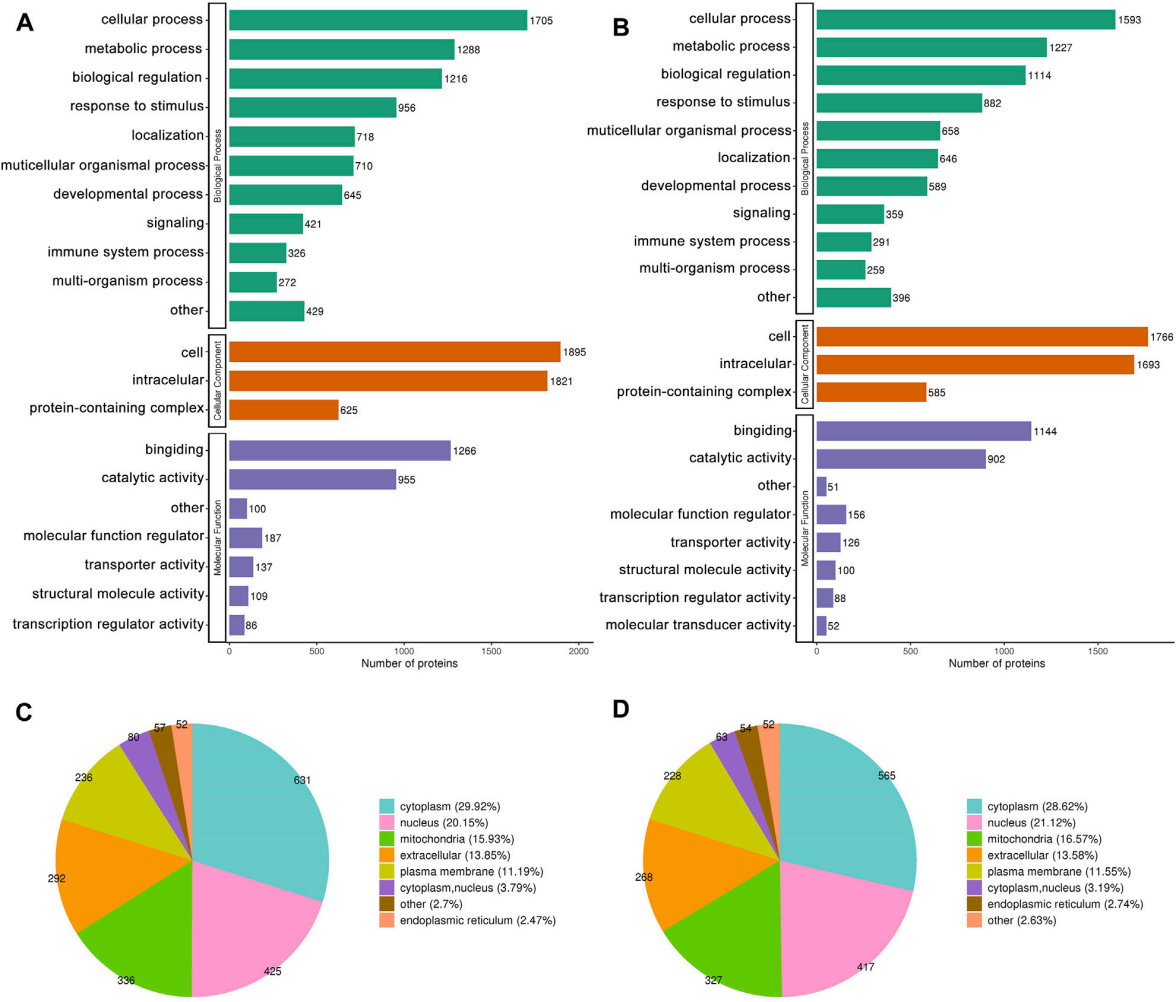
GO Annotation Classification and subcellular localization of DEPs. **(A,C)** Between MOD and CON groups, **(B,D)** between GPs and MOD groups.

Figures 3A, B depict the distribution of DEPs in GO annotations, including BP, MF, and CC, was analyzed to understand the biological functions of proteins from various viewpoints. The DEPs in both the MOD and GPs groups were associated with cellular and metabolic processes, biological regulation, and stimulus responses through BP annotation. In terms of MF, they mainly participate in binding and catalytic activity. For CC, DEPs were derived from cellular, intracellular, and protein-containing complexes. The subcellular structure location results showed that the DEPs in both MOD and GPs groups were localized in the cytoplasm, nucleus, mitochondria, and extracellular (Figures 3C, D).

**FIGURE 4:**
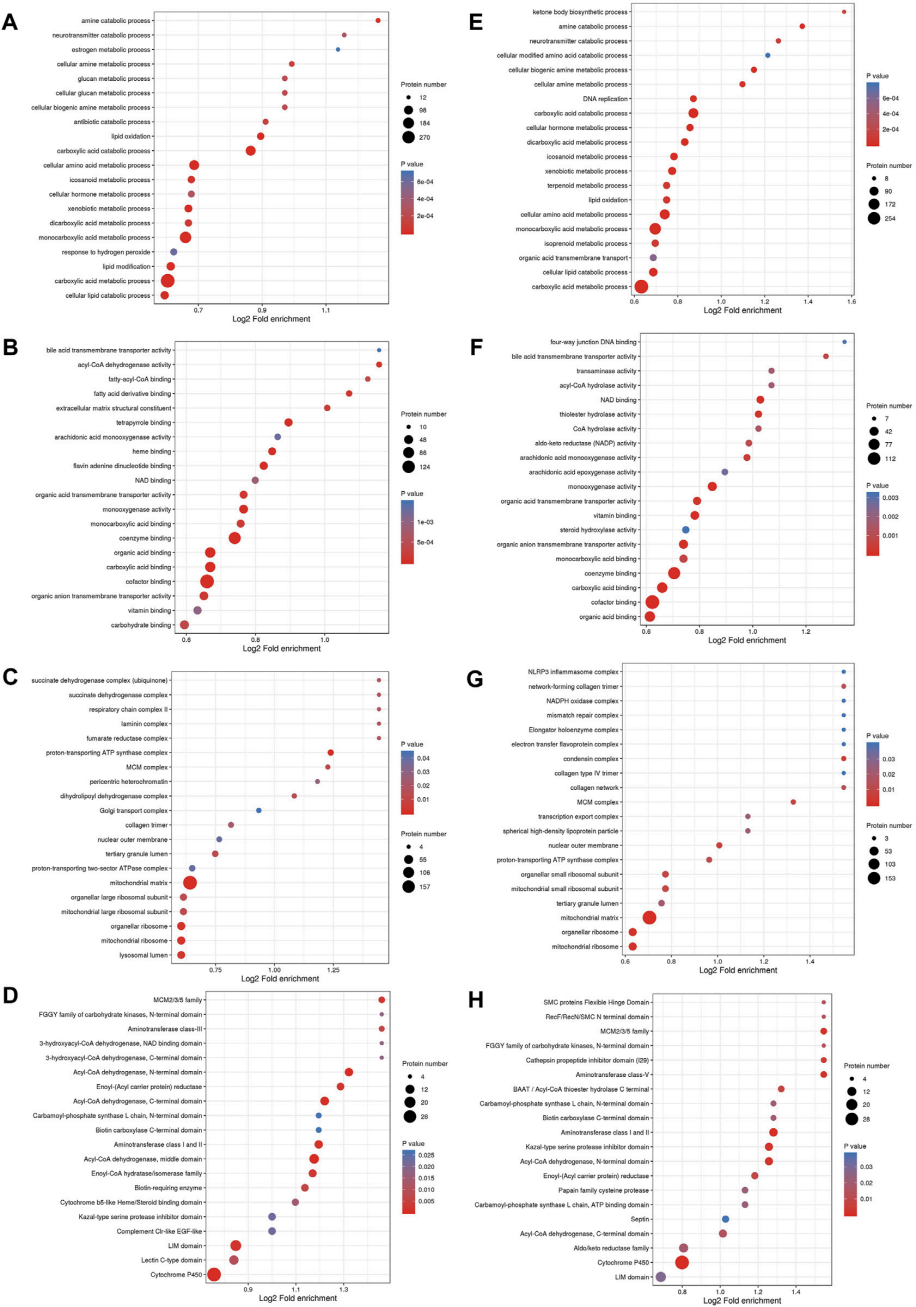
Functional enrichment analysis of DEPs. Bubble diagram of enrichment of DEPs between MOD and CON groups, **(A)** BP, **(B)** MF, **(C)** CC, **(D)** Protein domain; between GPs and MOD groups, **(E)** BP, **(F)** MF, **(G)** CC, **(H)** Protein domain. The longitudinal axis of the bubble diagram represents the functional classification, and the horizontal axis represents the Log2 transformed value of the proportion of DEPs. The circle’s color represents the *p*-value of enrichment significance, and its size represents the number of DEPs.

GO enrichment analysis was conducted to identify enrichment trends of DEPs in specific functional groups. For DEPs in both the MOD and GPs groups, BP enrichment indicated that proteins were mainly involved in carboxylic acid metabolic processes, carboxylic acid catabolic processes, and monocarboxylic acid metabolic processes (Figures 4A, E). MF enrichment showed that the DEPs were related to cofactor and coenzyme binding (Figures 4B, F). In CC, DEPs were associated with the mitochondrial matrix (Figures 4C, G). Protein domain enrichment revealed that the DEPs were involved in Cytochrome P450 (Figures 4D, H).

#### 3.3.2 KEGG pathways of DEPs

**FIGURE 5:**
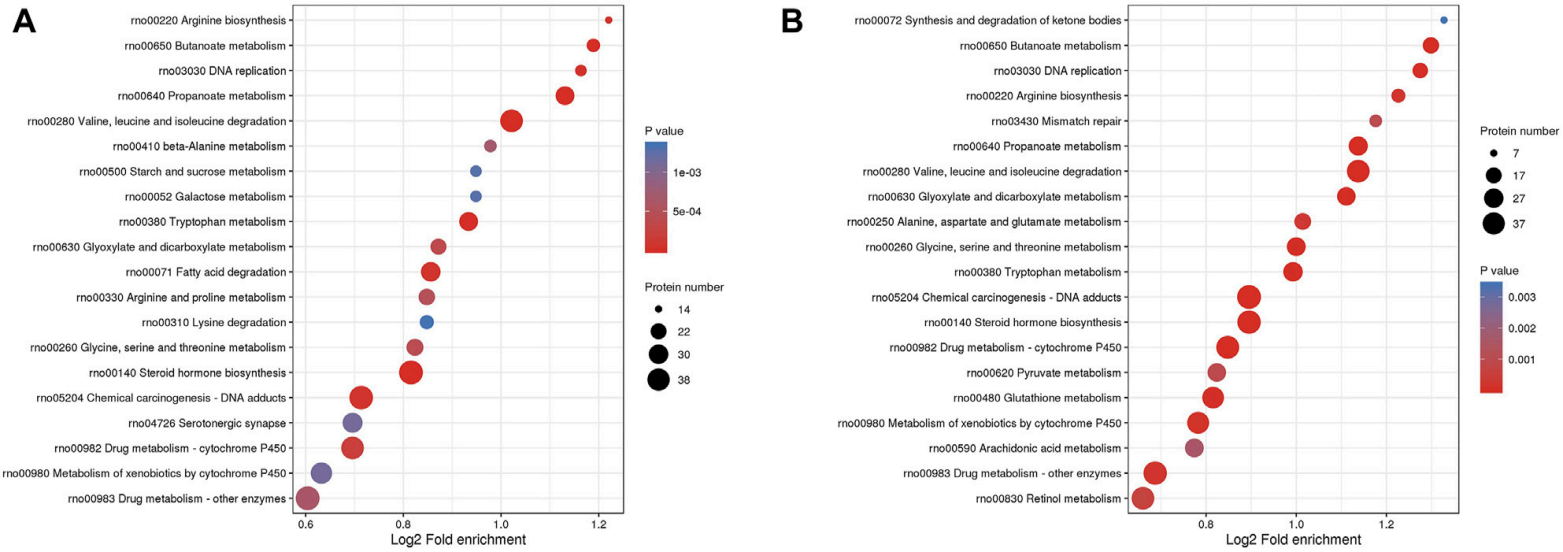
The bubble plot of KEGG enrichment analysis. **(A)** between MOD and CON groups, **(B)** between GPs and MOD groups.

KEGG enrichment analysis identified 37 relevant pathways (*p* < 0.05) between the MOD and CON groups. The analysis highlighted pathways such as the degradation of valine, leucine, and isoleucine; the metabolism of propanoate; steroid hormone biosynthesis and butanoate metabolism were primarily involved in the DEPs between the MOD and CON groups (Figure 5A). Additionally, 40 pathways were significantly associated (*p* < 0.05) with the action of GPs against liver injury. DEPs for GPs treatment of liver injury focus on the same pathways described above (Figure 5B). The full list of KEGG pathways is available in Supplementary Tables S3, S4.

### 3.4 Validation of lable-free data for selected proteins by PRM

**FIGURE 6:**
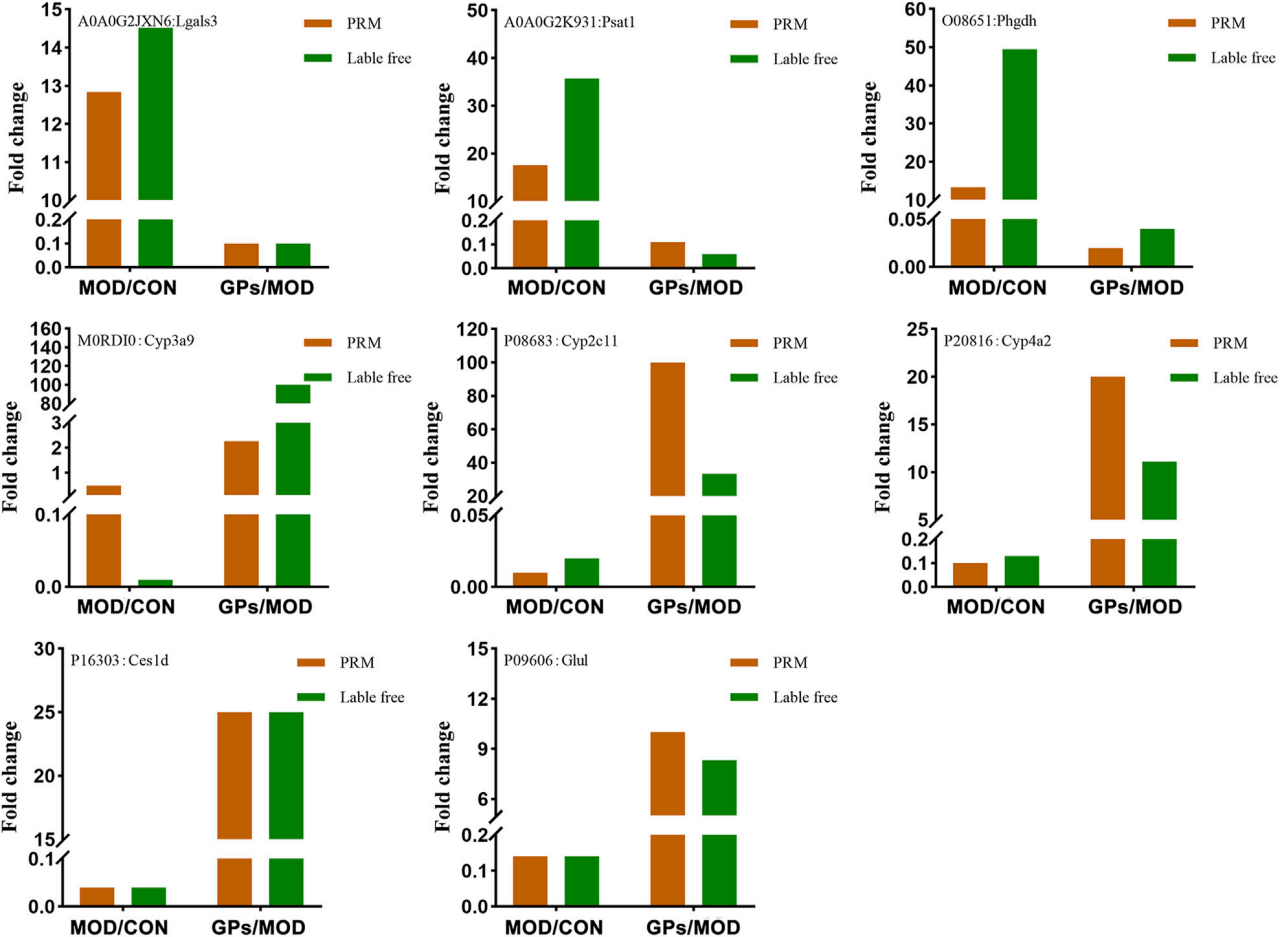
Relative expression levels of selected proteins measured via PRM.

Lgals3, Psat1, Cyp3a9, Phgdh, Cyp2c11, Glul, Ces1d, and Cyp4a2, were selected for validation, based on the FC values (provided in Supplementary Tables S1, S2) and related literature. Among these, Lgals3, Psat1, and Phgdh were increased in liver-injured tissues, while the levels of Cyp3a9, Cyp2c11, Cyp4a2, Glul, and Ces1d were decreased (Figure 6). Following GPs treatment, these levels were reversed, which is consistent with the label-free results. Proteomic functional analysis revealed that the eight DEPs, except Lgals3, perform catalytic molecular function. Psat1 and Phgdh are involved in glycine, serine, and threonine metabolisms, respectively. Cyp3a9, Cyp4a2, and Cyp2c11 are involved in chemical carcinogenesis and inflammatory mediator regulation. Glul is involved in glutamate metabolism, and Ces1d is involved in drug metabolism.

## 4 Discussion

The increasing prevalence of the use of active plant compounds for the treatment of clinical diseases necessitate the evaluation of their efficacy and mechanisms. Previous studies have shown that GPs may reduce collagen content, inhibit liver fibrosis (Chen et al., 2000), protect against ischemia/reperfusion-induced hepatic injury (Zhao et al., 2014), and treat fatty liver disease (Qin et al., 2012). This study observed a significant decrease in the expression of ALT and AST in the liver, which confirmed the protective effect of GPs on hepatocytes. Furthermore, the findings on liver and spleen indices and the results of HE staining illustrate the hepatoprotective and immunomodulatory effects of GPs.

Based on the metabolic pathways through which GPs exert their therapeutic effects, as elucidated by previous multi-omics studies, we further investigated the targets of GPs by integrating multiple proteomics technologies. We utilized the wide dynamic range of label-free technologies to identify low-abundance proteins and elucidated the functions of DEPs through bioinformatics analysis. Additionally, PRM technology was employed to validate some of the screened proteins, thereby addressing the limitation of the quantitative capability inherent in label-free technology.

First, the results of bioinformatics analysis further elucidated the mechanisms identified in previous experiments, which involve butanoate metabolism, glycine, serine, and threonine metabolism, the synthesis and degradation of ketone bodies, and phenylalanine metabolism (Tian et al., 2022). Using proteomics, protein targets of GPs related to these metabolic pathways were identified (Supplementary Table S4). Additionally, this study demonstrated that CCl_4_ induces deficiencies in branched-chain amino acids (BCAAs), disturbances in short-chain fatty acid metabolism, and abnormalities in steroid hormone biosynthesis (Gambella et al., 1992; Kawaguchi et al., 2014; Wang et al., 2022). And found for the first time that GPs can regulate branched chain amino acids metabolism and steroid hormone biosynthesis. This comprehensive analysis provides a deeper understanding of the molecular mechanisms underlying the hepatoprotective effects of GPs.

To determine the reliability of these protein levels, we selected eight proteins for quantitative PRM analysis, further confirming that they could serve as potential biomarkers and therapeutic targets. Cyp3a9 (Uniprot ID: M0RDI0), Cyp2c11 (Uniprot ID: P08683), and Cyp4a2 (Uniprot ID: P20816) belong to the cytochrome P450 (CYP450) family. CCl_4_ can be converted to the hepatotoxic substance trichloromethyl radical (CCl3·) by CYP450 enzymes, and then forms peroxidative trichloromethyl radical (CCl3OO·), which triggers increased lipid peroxidation, mitochondrial damage, and apoptosis of hepatocytes. Consequently, CCl_4_-induced liver injury results in a significant reduction in the activity and protein expression of major liver P450 isoenzymes (Xie et al., 2014). In this study, the levels of the three proteins were found to be decreased in CCl_4_-induced liver injury, and DEPs were predominantly enriched in the protein structural domain of CYP450, indicating the CCl_4_-induced liver injury was successfully established. CYP3A9 and CYP2C11 are homologs of the human liver enzymes CYP3A4 and CYP2C9, respectively. These enzymes play a crucial role in the metabolism of toxins and a wide range of drugs. Studies have demonstrated that inhibition of CYP3A4 can result in increased liver injury (Xiao et al., 2020). However, CYP4a2 expression is often positively correlated with lipid peroxidation (Song et al., 2023). Thus, it is hypothesized that CCl_4_ could inhibit the expression of most CYP450 enzymes, whereas GPs could restore the expression of these enzymes, which is supported by the proteomics results presented in Supplementary Tables S1, S2.

Lgals3 (UniProt ID: A0A0G2JXN6), galectin, is the most prominent galactose lectin in the disease. Previous studies have shown that galectin is associated with the physiopathology of various chronic liver disease (Jeng et al., 1994; Sano et al., 2000). Currently, galectin is considered a potential target for the early treatment of many liver diseases as it directly activates NLRP3 inflammatory vesicles, which play a key role in liver fibrosis (Mackinnon et al., 2023). Psat1 (Uniprot ID: A0A0G2K931) and Phgdh (Uniprot ID: O08651) are critical enzymes involved in the *de novo* synthesis of serine, directing the glycolytic intermediate 3-phosphoglyceric acid into the pathway. Recent studies have revealed that in hepatocellular carcinoma, PHGDH has been found to increase histone methylation levels and facilitate the transition from glycolysis to serine biosynthesis, thereby accelerating the development of hepatocellular carcinoma (Liu et al., 2017). In addition, serine synthesis (including *PHGDH* expression) is increased in the lungs of patients with pulmonary fibrosis to promote collagen fiber synthesis (Nigdelioglu et al., 2016). In this study, it was observed that the expression levels of Lgals3, Psat1, and Phgdh were elevated in rats with liver injury induced by CCl_4_, indicating an augmentation in collagen fiber levels in the liver, consistent with our previous study (Zhang et al., 2021b). It has been shown that GPs can significantly reduce CCl_4_-induced hepatic collagen deposition in rats, thereby ameliorating hepatic fibrosis (Chen et al., 2017). Therefore, the reduction in Lgals3, Psat1, and Phgdh levels observed in the rat liver following treatment with GPs indicates a potential mechanism whereby GPs influenced these targets, which may indirectly impact NLRP3 inflammatory vesicles and diminish *de novo* serine synthesis. This process could lead to a reduction in hepatic collagen deposition for treating liver injury.

Ces1d (Uniprot ID: P16303) is a carboxylesterase involved in hepatic fat mobilization, with triglyceride hydrolysis activity and the ability. Ces1d deficiency leads to disturbed glucose and lipid metabolism in both adipose tissue and liver (Xu et al., 2014). GPs demonstrate significant hypolipidemic and hypoglycemic effects and are widely employed in clinical treatment. Mechanistic studies have revealed that GPs play a pivotal role in reducing triglyceride levels and restoring lipid metabolism in hyperlipidemic mice (Xie J. B. et al., 2023). Furthermore, it has been found to restore sphingolipid and glycerophospholipid metabolism in mice with glucose metabolism disorders (Xie P. et al., 2023). In conjunction with this study, which showed that GPs elevate the expression of hepatic Ces1d in liver-injured rats, indicating that GPs may restore glucose and lipid metabolism by modulating CES1.

Glul (Uniprot ID: P09606), glutamine synthetase, converts glutamate and ammonia to glutamine, which is the main pathway for ammonia transport and storage and is essential for maintaining nitrogen homeostasis. Studies have shown that remodeling of glutamine and ammonia metabolism mediated by hepatocellular glutamine synthetase effectively alleviates hepatic fibrosis (Wu et al., 2022). Dysfunction of glutamine synthetase in damaged hepatocytes leads to accumulation of harmful ammonia in the blood, which results in hepatic encephalopathy. In this study, the level of Glul decreased in rats with liver injury, consistent with previous studies (Schmidt et al., 2021). The level of Glul was reversed after GPs treatment, indicating that GPs may restore glutamine and ammonia metabolism in the treatment of liver injury.

## 5 Conclusion

In conclusion, protein profiles were established through label-free proteomics, and bioinformatics analysis showed that GPs mainly restored the degradation of valine, leucine, and isoleucine, as well as propanoate and butanoate metabolism, and steroid hormone biosynthesis during liver injury. Meanwhile, 1,508 DEPs related to the hepatoprotective effects of GPs were identified, followed by PRM validation, eight target proteins of GPs were identified for the first time, including Lgals3, Psat1, Phgdh, Cyp3a9, Cyp2c11, Cyp4a2, Glul, and Ces1d. These proteins enhance our understanding of the therapeutic mechanisms of GPs and may also be candidate therapeutic targets for chronic liver disease, providing molecular evidence for chronic liver disease research.

## Data Availability

The data presented in the study are deposited in the iProX repository, accession number IPX0008035001 and IPX0008036001.
