# Supplementary material for: Label-free proteomic analysis reveals the hepatoprotective mechanism of gypenosides in liver injury rats
Source: Front Pharmacol. 2024 Jun 27;15:1417575. doi: 10.3389/fphar.2024.1417575 (PMC11236725; doi:10.3389/fphar.2024.1417575)
Supplement: Supplementary file 5 [file DataSheet1.docx]

Supplementary Material

# Supplementary Data

# Supplementary Figures and Tables

## Supplementary Figures

(B)

(A)


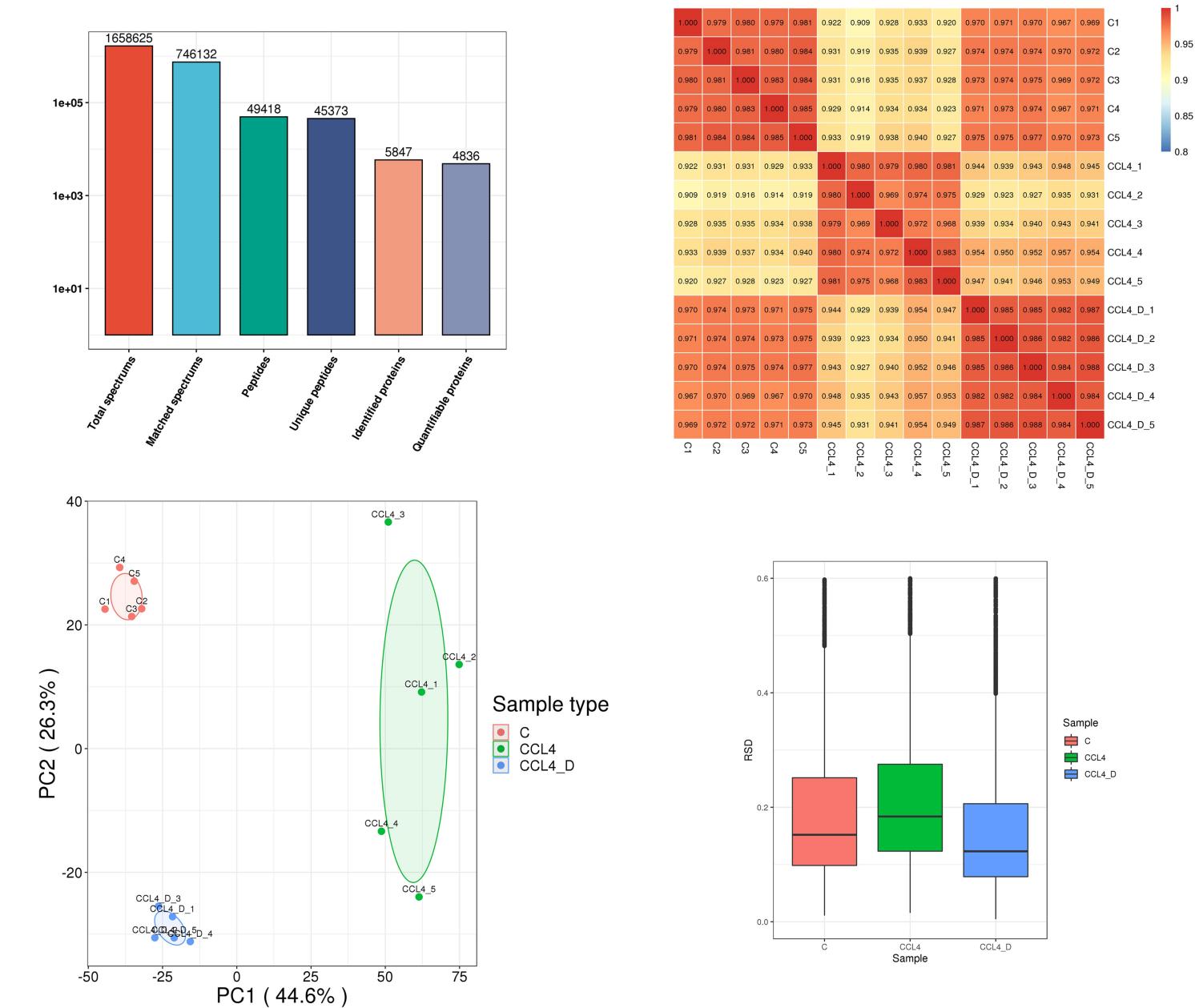


(D)

(C)

**Supplementary Figure 1.** Statistical analysis of identified proteins. (A) Overview of protein identification; (B) Pearson correlation coefficient; (C) Principal component analysis; (D) Relative standard deviation.
